# Case Report: An overlooked inverted supernumerary tooth: a rare presentation with nasal invasion and its diagnostic and therapeutic implications

**DOI:** 10.3389/fmed.2026.1784219

**Published:** 2026-04-07

**Authors:** Yuting Lv, Ruofei Xu, Tingting Fang, Yumin Mao

**Affiliations:** 1 Dentistry Department, Longyou County Traditional Chinese Medicine Hospital, Longyou, China; 2 Orthopedics Department, Longyou County People's Hospital, Longyou, China; 3 Dermatology Department, Longyou County People's Hospital, Longyou, China; 4 Dentistry Department, Wangdian People's Hospital, Jiaxing, China

**Keywords:** cone-beam computed tomography, inverted, minimally invasive surgery, nasal symptoms, supernumerary tooth

## Abstract

**Background:**

Supernumerary teeth (STs) are common odontogenic anomalies, while inverted STs with palatal perforation and nasal invasion are extremely rare and carry a high risk of misdiagnosis. This case highlights the value of interdisciplinary awareness and advanced imaging.

**Case Introduction:**

A 9-year-old boy with unilateral nasal obstruction (previously misdiagnosed as rhinitis) was referred to dentistry due to dental trauma. Initial panoramic X-ray identified an inverted ST but lacked clarity. Subsequent cone-beam computed tomography (CBCT) with multiplanar reconstruction and 3D segmentation confirmed an inverted ST in the anterior maxilla with palatal perforation and nasal invasion. The tooth was extracted via a minimally invasive palatal approach guided by a 3D model, resulting in immediate resolution of nasal symptoms and a good postoperative recovery.

**Conclusion:**

Odontogenic causes must be included in the differential diagnosis for children with refractory unilateral nasal symptoms, highlighting the critical need for interdisciplinary collaboration between dentists and otorhinolaryngologists. While panoramic X-ray is a useful preliminary screening tool, CBCT is essential—not only for accurate preoperative diagnosis and safe, minimally invasive surgical planning but also for postoperative confirmation of complete removal.

## 1 Introduction

Supernumerary teeth (STs) are common dental anomalies. They are defined as teeth or tooth-like structures that exceed the normal 20 primary or 32 permanent teeth (1). The anterior maxilla is the most common site, but STs can also appear in the mandible, nasal cavity, and maxillary sinus (2–5). ST prevalence in the general population ranges from about 0.3% to 3.5%. They are seen more often in permanent teeth, with a clear preference for males (2, 3). Inverted impaction, where the crown points apically, is rare but clinically important. In the anterior maxilla, inverted STs sometimes migrate and invade nearby structures, such as the nasal cavity (6, 7). When they cause palatal perforation or nasal invasion, patients may show unilateral nasal obstruction or discharge. This can mimic rhinitis or sinusitis and may be misdiagnosed by otorhinolaryngologists (8–10). Recent cone-beam computed tomography (CBCT) studies have improved diagnosis. They show that ectopic teeth often have complex three-dimensional positions not fully seen with standard two-dimensional imaging (2, 11).

The pathogenesis of ST is not fully understood and is widely considered to result from abnormal tooth developmental programming due to combined genetic and environmental factors. Classic etiological hypotheses include dichotomy of the tooth germ, hyperactivity of the dental lamina, atavism, and the widely observed genetic predisposition (12, 13). However, these macroscopic theories require a molecular-level explanation. Recent research reveals that normal tooth development relies on a precisely regulated signaling network between the dental epithelium and mesenchyme. Key pathways, including Wnt/β-catenin, Sonic Hedgehog (SHH), Fibroblast Growth Factor (FGF), and Bone Morphogenetic Protein (BMP), collectively determine tooth germ formation, positioning, morphogenesis, and differentiation (14, 15).

This case report details the diagnosis and management of a completely inverted, nasally invading ST in a child. It highlights the pivotal role of CBCT in diagnosis and surgical planning after initial screening with panoramic X-ray, and discusses the etiology, diagnostic imaging, and principles of minimally invasive surgery for complex STs.

## 2 Case report

### 2.1 Patient information

A 9-year-old male patient presented to the Department of Stomatology with a chief complaint of traumatic injury to the maxillary anterior teeth. During history taking, the patient also reported persistent right-sided nasal obstruction and occasional dull nasal pain for 1 month. There was no history of purulent discharge, fever, or epistaxis. The nasal symptoms had previously been diagnosed as allergic rhinitis by an otorhinolaryngologist, and treatment with mometasone furoate nasal spray once daily was initiated. Although symptoms showed slight improvement, they did not resolve completely. The patient's medical and dental history was otherwise unremarkable, with no known allergies or family history of supernumerary teeth.

### 2.2 Clinical examination

Extraoral Examination: Facial symmetry was normal, with adequate mouth opening and no palpable cervical lymphadenopathy.

Intraoral Examination: Mixed dentition stage. Teeth from the maxillary right canine to the maxillary left lateral incisor were erupted. Tooth 21 presented with an enamel-only crown fracture, no pulp exposure, no sensitivity on probing, and no abnormal mobility. Tooth 11 was intact and non-tender to percussion. The mucosa covering the palatal and labial aspects of the anterior maxilla appeared normal in color, texture, and contour, with no swelling, erythema, or fistula. Mild crowding (Grade I) was noted in both arches.

### 2.3 Diagnostic assessment

**Figure 1:**
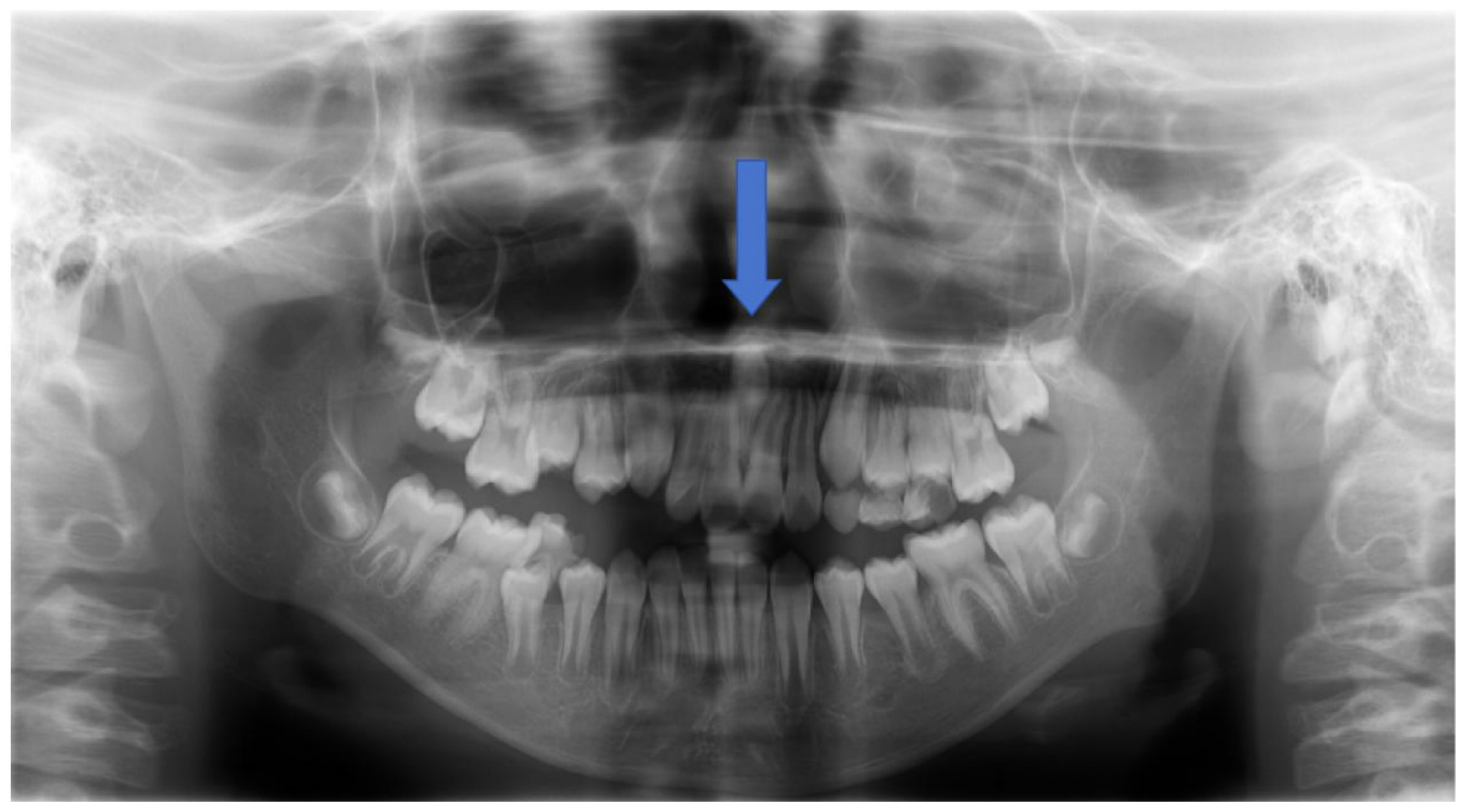
Panoramic radiograph demonstrating the inverted supernumerary tooth in the anterior maxilla (indicated by a blue arrow).

Upon admission for the maxillary anterior tooth fracture, a panoramic X-ray was first performed as a routine screening examination to evaluate the extent of the dental trauma and the overall dental status (Figure 1). This initial panoramic X-ray identified the presence of an inverted supernumerary tooth in the anterior maxilla, but due to the limitations of two-dimensional imaging—including anatomical structure superimposition and insufficient spatial resolution—the detailed orientation of the tooth, the extent of its impaction, and its relationship with the hard palate, nasal cavity, and adjacent permanent teeth could not be clearly visualized.

To further clarify the lesion and facilitate subsequent treatment planning, CBCT was performed. Images were acquired using a CLINVIEW 11.9.1 CBCT scanner (Instrumentarium Dental, Tuusula, Finland) with the following parameters: field of view (FOV) 8 cm × 8 cm, voxel size 0.318 mm, and exposure settings of 90 kV, 10 mA, and 12 s. Image analysis was conducted using OnDemand3D Dental software (Cybermed Inc., Seoul, Korea) with a window level (WL) set to 583, a window width (WW) to 3,144, and a zoom factor of × 0.8.

**Figure 2:**
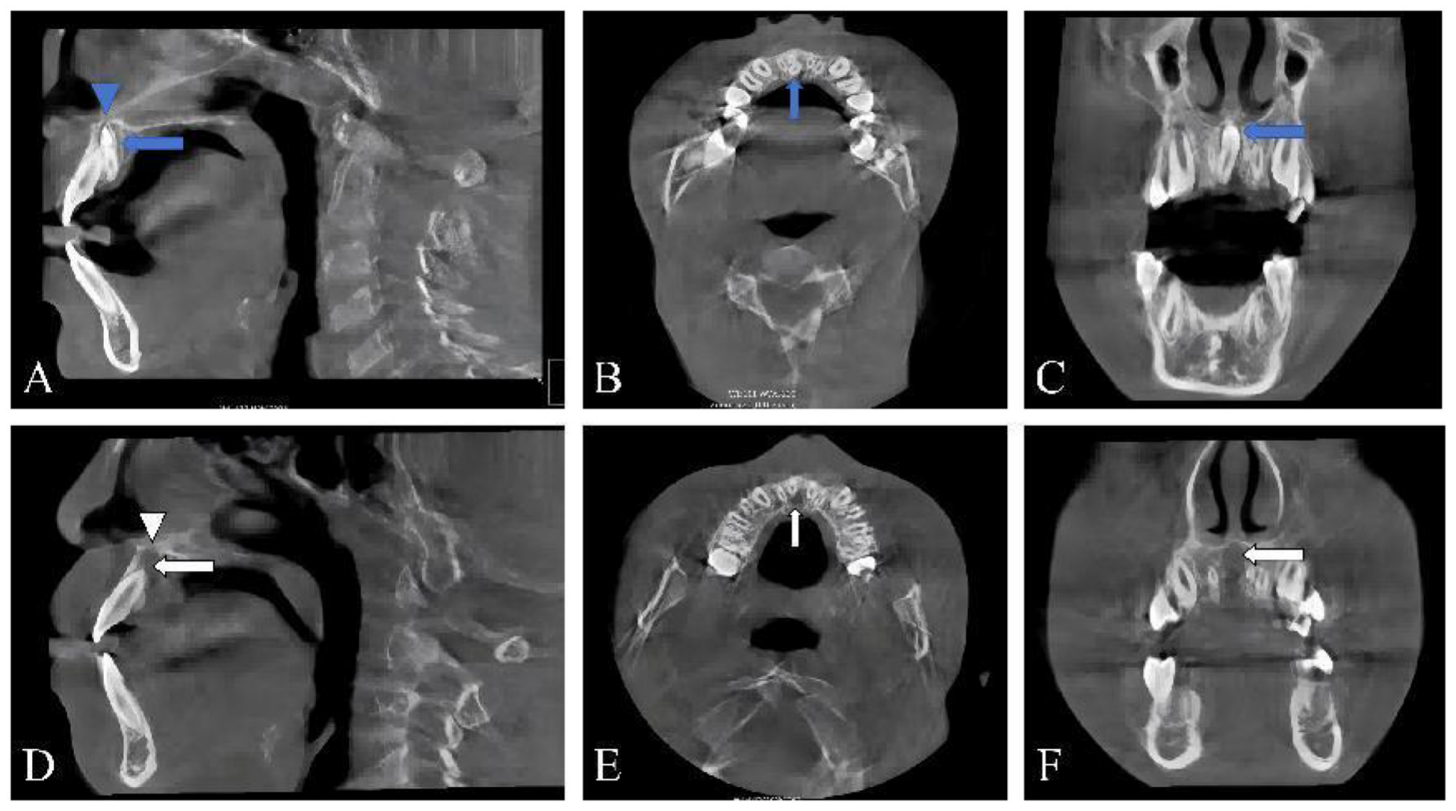
Preoperative and postoperative CBCT images of the inverted supernumerary tooth in the maxilla. **(A–C)** Preoperative images clearly demonstrate the location of the supernumerary tooth (indicated by blue arrows). **(A)** Sagittal view shows perforation of the hard palate by the tooth crown (indicated by a blue triangle). **(B)** Axial view reveals the close proximity between the root of the supernumerary tooth and the adjacent tooth root. **(C)** Coronal view confirms the completely inverted orientation of the tooth. **(D–F)** Postoperative images confirm the complete removal of the supernumerary tooth. The original site is now visible as a well-defined extraction socket (indicated by white arrows). A corresponding bony defect is evident at the site of the previous palatal perforation (indicated by a white triangle).

Multiplanar reconstruction in orthogonal views (sagittal, coronal, and axial) was employed for primary diagnostic interpretation. Interactive slice navigation enabled precise assessment of the tooth's orientation and its spatial relationship with adjacent anatomical structures. Specific imaging findings were as follows:

Sagittal View (Figure 2A): Confirmed the ST's location slightly left of the midline. Crucially, the image showed the crown had completely perforated the cortical bone of the hard palate. A focal hypodense area adjacent to the crown tip near the nasal mucosa suggested follicular tissue, possibly with mild inflammation.

Axial View (Figure 2B): Demonstrated the close relationship between the ST's root and the root surface of tooth 21, as well as its proximity to the nasopalatine canal.

Coronal View (Figure 2C): Revealed a completely inverted, hyperdense, impacted tooth. The crown pointed superiorly toward the nasal cavity, overlapping the hard palate, while the root apex pointed inferiorly, terminating approximately 3 mm apical to the crestal bone in the edentulous area adjacent to tooth 21.

Using anatomical landmarks (incisive foramen, nasal floor, hard palate, tooth 21 root), the ST was precisely localized. The diagnosis was confirmed as an inverted supernumerary tooth in the anterior maxilla with palatal perforation and nasal invasion.

### 2.4 Treatment intervention

Considering the family's strong preference for minimally invasive surgery and high esthetic demands, along with CBCT findings indicating that the lowest point of the supernumerary tooth apex was located approximately 3 mm below the alveolar crest of tooth 21, a palatal intrasulcular incision approach was selected. The surgery was performed by an experienced oral and maxillofacial surgeon. This approach was chosen to optimize blood supply to the surgical site, preserve the interdental papilla, and achieve the desired minimally invasive outcome. Following local infiltration anesthesia, an incision was made along the palatal cervical margins of teeth 13 to 23 using a No. 3 scalpel handle with a No. 15 blade, severing the epithelial attachment down to the bone surface, and a full-thickness mucoperiosteal flap was elevated. Soft tissues were gently retracted throughout the procedure to avoid any undue manipulation. Guided by CBCT imaging, a piezosurgery unit was used to create a bony window at the corresponding site, exposing the root of the ST. The osteotomy procedure was completed in approximately 15 min. A minimally invasive elevator was then inserted, and gentle rotational movements were applied to create a sufficient extraction pathway. Care was taken to strictly avoid applying any apical force to prevent displacement of the tooth. Once adequately luxated, the supernumerary tooth was extracted in toto. The follicular sac was thoroughly curetted, the surgical site was copiously irrigated with normal saline, and the mucoperiosteal flap was repositioned. Closure was achieved using interrupted sutures with 5-0 non-resorbable suture material. Postoperatively, the patient was prescribed a course of cephalosporin antibiotics to prevent infection and ibuprofen for pain management.

### 2.5 Follow-up and outcome

The patient reported complete resolution of nasal obstruction and pain from the first postoperative day. A standardized follow-up protocol was implemented. At the 1-week postoperative visit, sutures were removed. During this visit, a postoperative CBCT scan was performed to assess the surgical site for potential complications, specifically to rule out any residual root fragments and to evaluate the integrity of the nasal floor, confirming no iatrogenic perforation or damage. The scan confirmed the complete removal of the ST (Figures 2D-F) and an intact nasal floor. The surgical site showed excellent primary healing. The morphology, color, and texture of the palatal gingiva were preserved, with no recession of interdental papillae or visible scarring. Subsequent follow-ups were scheduled at 1 month and 3 months postoperatively to monitor mucosal healing, evaluate the vitality and stability of the adjacent teeth (particularly tooth 21), and assess for any recurrence of nasal symptoms. The patient remained asymptomatic during all reviews, and the adjacent teeth responded normally to vitality testing.

## 3 Discussion

Although STs are a common developmental anomaly, their complete inversion with nasal symptoms is exceedingly rare. This case is unique as it provides a template for leveraging advances in diagnostic technology and precision surgery to manage complex ectopic eruptions.

Beyond the nasal invasion and palatal perforation observed here, STs typically cause local oral complications such as impaction of permanent teeth, dentigerous cysts, and occlusal disturbances (16–18). In rare instances, STs invade the nasal cavity, where their nonspecific symptoms often mimic rhinitis, leading to frequent misdiagnosis (19).

### 3.1 Paradigm shift in imaging diagnosis: from two-dimensional planes to multiplanar reconstruction and precision localization

The diagnosis and localization of ST heavily depend on radiographic examination. Traditional two-dimensional techniques, such as panoramic and periapical radiographs, remain valuable as initial diagnostic tools. However, they have inherent limitations, including superimposition and unclear display of anatomical structures (11). These limitations are particularly pronounced when evaluating deeply impacted, inverted, or ectopic STs in special locations like the nasal cavity or maxillary sinus, often leading to missed diagnosis or misjudgment.

CBCT overcomes these limitations and has become a crucial diagnostic aid in contemporary management protocols (2). Compared to conventional CT, CBCT offers advantages of lower radiation dose, higher spatial resolution, and shorter scan time (20). It provides isotropic three-dimensional data, enabling multiplanar reconstruction (sagittal, coronal, and axial) and three-dimensional volume rendering (21). This represents a leap in diagnostic capability: it not only confirms the presence of an ST but also precisely and three-dimensionally visualizes its inverted morphology, the exact location and extent of palatal perforation, the millimetric distance to adjacent tooth roots (e.g., tooth 21), and the relationship to vital structures like the nasopalatine canal. This indispensable spatial information, derived from multiplanar reconstruction, is entirely unattainable with traditional 2D imaging. The “surgical map” provided by CBCT forms the basis for accurate risk assessment and preoperative planning, which is the prerequisite for achieving minimally invasive and safe surgery.

### 3.2 CBCT-guided precision minimally invasive surgical practice

The therapeutic success of this case exemplifies the principle of “precision diagnosis guiding precision treatment” (22, 23). The core of this approach lies in the systematic translation of multiplanar CBCT data into clinical decisions at each critical surgical stage, operationalized through three key steps. First, personalized access design based on three-dimensional visualization. CBCT with multiplanar reconstruction confirmed the tooth's complete inversion (crown toward nasal floor, root apically positioned). This precise spatial information guided the selection of a minimally invasive palatal intrasulcular approach, providing direct access to the root while preserving the aesthetics and blood supply of the palatal gingival papillae, consistent with modern periodontal microsurgery principles (24). Second, intraoperative navigation using quantitative CBCT data. The CBCT dataset served as a preoperative blueprint, allowing precise determination of the optimal osteotomy window location, dimensions, and depth on multiplanar images. Intraoperatively, piezoelectric surgery enabled bone removal strictly within this pre-planned window, transforming the procedure from spatial estimation into data-guided navigation. This minimized injury risk to adjacent structures (tooth 21 root, nasopalatine nerve) and reduced unnecessary bone removal (25). Third, biomechanically sound execution informed by anatomical risk assessment. Preoperative CBCT analysis of the spatial relationship between the tooth root, nasal floor, and palatal bone plate enabled accurate prediction of withdrawal resistance. This informed the core operative principle of strictly avoiding apically directed forces. During surgery, rotational and traction forces were applied instead of axial impact, ensuring safe luxation toward the oral cavity along a pre-determined path and effectively preventing iatrogenic displacement into the nasal cavity (26).

### 3.3 Clinical implications: interdisciplinary thinking and differential diagnosis of rare symptoms

The diagnostic trajectory of this case demonstrates that odontogenic lesions originating from the jaws but invading the nasal cavity can present solely as dysfunction of the adjacent organ (e.g., nasal obstruction and pain in this case), leading to initial consultation with otorhinolaryngologists and potential misdiagnosis as primary rhinitis. This case was ultimately managed through a coordinated interdisciplinary approach: the otorhinolaryngologist initially evaluated the nasal symptoms, the dentist identified the ectopic tooth on a panoramic radiograph obtained for trauma, and the oral and maxillofacial surgeon performed the definitive diagnosis and treatment using CBCT and minimally invasive surgery. Therefore, for unexplained, treatment-resistant unilateral nasal symptoms, especially in pediatric patients, clinicians (including both dentists and otorhinolaryngologists) should adopt an interdisciplinary mindset and actively include odontogenic pathology in the differential diagnosis. Prompt maxillofacial imaging (e.g., CBCT) is key to achieving a definitive diagnosis and avoiding delays.

#### 3.3.1 Limitations

This is a single case report. Although it comprehensively illustrates the entire diagnostic and therapeutic process of such a rare entity, the generalizability of its conclusions requires validation through the accumulation of more cases and long-term follow-up studies.

## 4 Conclusion

In summary, this case report describes a rare, completely inverted supernumerary tooth presenting with maxillary nasal symptoms. Through this case, we have highlighted the indispensable core value of CBCT multiplanar reconstruction in diagnosing complex dentomaxillofacial pathologies and demonstrated the successful application of individualized, precision-based minimally invasive surgery. This case suggests that when confronted with atypical craniofacial symptoms, the differential diagnostic spectrum should be broadened, interdisciplinary collaboration strengthened, and multiplanar reconstruction imaging technology fully utilized to achieve early diagnosis and precise treatment.

## Data Availability

The original contributions presented in the study are included in the article/supplementary material, further inquiries can be directed to the corresponding author.
